# Draft Genome Sequence of *Sclerotinia borealis*, a Psychrophilic Plant Pathogenic Fungus

**DOI:** 10.1128/genomeA.01175-13

**Published:** 2014-01-23

**Authors:** Andrey V. Mardanov, Alexey V. Beletsky, Vitaly V. Kadnikov, Alexander N. Ignatov, Nikolai V. Ravin

**Affiliations:** Centre Bioengineering, Russian Academy of Sciences, Moscow, Russia

## Abstract

*Sclerotinia borealis* is a necrotrophic phytopathogenic fungus notable for its wide host range and environmental persistence. It grows at low temperatures, causing snow mold disease of crop plants. To understand the molecular mechanisms of its pathogenesis and adaptation to the psychrophilic lifestyle, we determined the 39.3-Mb draft genome sequence of *S. borealis* F-4128.

## GENOME ANNOUNCEMENT

*Sclerotinia* species are among the most diverse and widely distributed phytopathogenic fungi causing many economically important diseases of crop plants (1). *Sclerotinia borealis* Bubák & Vleugel has a broad host range, infecting at least 17 plant genera from the families *Alliaceae*, *Asteraceae*, *Brassicaceae*, *Campanulaceae*, *Fabaceae*, *Iridaceae*, *Pinaceae*, and *Poaceae*. This fungus is notable for being a psychrophile, with an optimum growth temperature between 4°C and 10°C (2, 3); it infects plants at quiescence under snow cover, causing important plant diseases in countries with cold climate (snow mold disease). *S. borealis* is a necrotroph; it first kills host plant cells and then colonizes the dead tissue.

The genome sequences of three taxonomically closely related strains of mesophilic phytopathogenic fungi, *Sclerotinia sclerotiorum* 1980, *Botrytis cinerea* T4, and *B. cinerea* B05.10, were sequenced previously (4). The genome sequences revealed a striking difference in the number and diversity of secondary metabolism genes, which may be involved in the adaptation of these strains to different ecological niches. To understand the molecular mechanisms of pathogenesis and adaptation to low temperatures, we determined the draft genome sequence of *S. borealis* F-4128.

*S. borealis* F-4128 was obtained from All-Russia Collection of Microorganisms (VKM). DNA was extracted using the modified method described in Möller et al. (5). The genome of *S. borealis* was sequenced using the Roche GS FLX pyrosequencing platform. Sequencing was performed using a whole-genome strategy employing shotgun and paired-end genome libraries. We obtained 2,148,202 shotgun and 494,835 paired-end reads; they were assembled into 1,256 scaffolds by the Newbler assembler 2.8 (454 Life Sciences, Branford, CT). The total size of the assembled genome of *S. borealis* is 39.3 Mb, with a G+C content of 42%, representing 23-fold coverage. *Ab initio* gene prediction was performed by using Augustus 2.7 (6) (http://bioinf.uni-greifswald.de/augustus/), producing 10,171 protein-coding sequences. The genomes of *S. borealis* and *S. sclerotiorium* are similar in size and show rather high sequence identity and local gene order conservation. The arsenal of genes associated with the necrotrophic lifestyle is also similar between species, including the genes involved in plant cell wall degradation. However, many genome regions and genes specific to *S. borealis* may be responsible for its adaptation to particular ecological niches and conditions of growth.

The genome sequence of *S. borealis* is a valuable recourse for identifying the genes for the central metabolic pathways and for analyses of the molecular mechanisms of pathogenesis and adaptation to grow at low temperatures. It will also serve as platform to facilitate comparative genomic studies involving psychrophilic pathogenic fungi, as well as other species in the order *Ascomycota*.

### Nucleotide sequence accession number.

The draft genome sequence of *S. borealis* F-4128 has been deposited in GenBank under the accession no. AYSA00000000. The version described in this article is the first version.
